# Household living conditions and mental health of youth and adults: differences between urban and rural regions

**DOI:** 10.1186/s40359-026-04906-7

**Published:** 2026-05-30

**Authors:** Laura Gorla, Jennifer E. Lansford, Arnab Mukherji, Marcos Vera-Hernández, Manoj Mohanan, Joanna Maselko

**Affiliations:** 1 Duke Center for Child and Family Policy, Duke University, Durham, USA; 2 Sanford School of Public Policy, Duke University, Durham, USA; 3 Center for Public Policy, Indian Institute of Management Bangalore, Bengaluru, India; 4 Department of Economics, University College London, London, UK; 5 Department of Economics, Duke University, Durham, USA; 6 Duke Global Health Institute, Duke University, Durham, USA; 7 Department of Epidemiology, Gillings School of Global Public Health, University of North Carolina, Chapel Hill, USA; 8 Carolina Population Center, University of North Carolina, Chapel Hill, USA

**Keywords:** Mental health, Urban and rural regions, Living conditions, Children, Adults

## Abstract

**Background:**

Among a broader set of household assets that support healthy living, access to water, a refrigerator, air conditioning, and domestic help may be especially relevant for mental health. However, these components of household living conditions vary substantially across regions and have rarely been examined in relation to mental health. This study examines how these key household living conditions relate to depression and anxiety among children and adolescents (i.e., youth) and adults, while also exploring urban and rural differences in these associations.

**Methods:**

Participants were interviewed in collaboration with the Centre for Monitoring Indian Economy (CMIE) that has been conducting a longitudinal survey covering 98% of India’s population. The current analysis is based on mental health data collected in June 2023, including 8,205 households living in rural regions and 19,979 households living in urban regions. These households were composed of 29,188 members in rural regions and 67,351 members in urban regions. Among these household members, 37,453 adults provided information on their own depression and anxiety, and 20,397 adults reported on the depression, and 19,363 on the anxiety of youth living in the household.

**Results:**

Overall, having unlimited access to water, a refrigerator, and domestic help was associated with lower depression and anxiety among youth and adults. Specifically, having a refrigerator and domestic help were related to lower depression and anxiety when individuals live in rural settings compared to urban ones, whereas air conditioning and water access were linked to lower depression and anxiety in urban settings.

**Conclusions:**

These findings highlight the connection between these living conditions and mental well-being, suggesting that interventions targeting mental health should be designed with the understanding that less access to water, refrigerator, air conditioning, and domestic help is related to poorer mental health.

**Supplementary Information:**

The online version contains supplementary material available at 10.1186/s40359-026-04906-7.

## Background

Article 25 of the Universal Declaration of Human Rights affirms that *“everyone has the right to a standard of living adequate for the health and well-being of himself and of his family*” [1]. This fundamental right, together with many others, is echoed in the Sustainable Development Goals (SDGs) 2015–2030 established by the United Nations as part of the 2030 Agenda for Sustainable Development [2], which aims to ensure that all individuals worldwide can realize their full potential in a healthy and supportive environment. Among all the several conditions necessary to uphold this right, living in a safe, clean, secure, and adequate home environment is essential to promote the physical and psychological health of youth and adults. However, in many parts of the world, such living conditions are still out of reach for millions. Moreover, although several studies have examined the adverse effects of inadequate living conditions on physical health, less attention has been devoted to understanding how these conditions influence individuals’ mental health, both among youth and adults.

Mental health is included in the SDGs, and growing evidence has pointed to a global mental health crisis (especially after the COVID-19 pandemic) [3, 4]. Worldwide, depression and anxiety are among the primary causes of illness and disability for approximately 8% of children aged 5–9 years and 15% of young people aged 10–19 years [5], and affect around 5% (depression) and 4% (anxiety) of adults [6]. Although more mental health support programs were created in the aftermath of COVID-19, significant difficulties persist in detecting and treating mental health problems in several areas of the world [5]. Given these numbers, advancing understanding of the elements associated with mental health across different life stages is timely and essential to advance targeted policies and interventions. Among the many aspects connected with mental health, household living conditions might be particularly important, as they shape individuals’ daily routines, experiences, opportunities, and their capacity to meet essential needs.

### Household living conditions and mental health: research findings and gaps

The strong link between household living conditions and individuals’ physical well-being is well-documented in the literature. Numerous studies have emphasized how housing quality, water and sanitation facilities, assets, and household amenities might promote or hinder physical health [7–9]. Poor and inadequate housing has been associated with a range of adverse outcomes, including stunting [10], underweight [11], low birth weight [12], diarrhea [13], infectious diseases [14], and mortality [15]. In exploring these topics, many studies operationalize living conditions as a type of housing material, therefore capturing basic shelter, while others focus on specific resources such as access to electricity. However, living conditions vary by region and are strongly related to the overall development of the area [16]. Moreover, among all the household infrastructures and assets that support healthy living, the literature on mental health has paid little attention to water, access to a refrigerator, air conditioning, and domestic help, underscoring the need for further research on this topic. These living conditions can be particularly important for sustaining the daily lives of individuals of different ages, as they ensure access to safe hydration and food preservation, facilitate more hygienic conditions, and protect against environmental stressors such as extreme heat or particularly demanding household chores and work. All these elements might significantly impact individuals’ well-being, making it crucial to explore the associations between water access, refrigerator ownership, air conditioning, domestic help, and mental health across different ages.

### Water access and mental health

Water access is an established determinant of physical health, essential for disease prevention, healthy nutrition, food security, gender equity, and economic development. Because water is used for drinking, cooking, and washing, access to a clean and adequate water source significantly reduces infections, unhygienic conditions, and mortality [17, 18]. Although the relationship between water and mental health has witnessed a surge in related studies over the last two decades, the literature on this topic remains less well-documented [19]. Available literature has highlighted a strong relation between water insecurity, physical difficulties, and emotional distress - especially for women [20], as well as social, material, and ecological factors associated with the lack of and insecurity of water access [21, 22]. For instance, water insecurity triggers everyday worries and uncertainties within the household members, is often associated with social stigma, raises worries about physical health, and might lead to family violence [23]. These findings have significant implications for public policy, requiring practitioners and decision-makers to incorporate mental health aspects into water access interventions and water management strategies [19].

### Refrigerators and mental health

The positive effects of owning a refrigerator, particularly in terms of hygiene, are quite clear. Refrigerators prevent food spoilage, which enables individuals to consume a wider variety of more nutrient-rich foods (such as fruits, vegetables, meat, and fish), not relying heavily on more processed, less nutritious foods that do not require refrigeration. Reducing food-related infections, contaminations and exposure to harmful pathogens [24], refrigerators also allow a family to purchase or prepare enough food for more than one meal, reducing food waste, saving money as well as time cooking and shopping. All these aspects contribute to the beneficial effects of owning a refrigerator on both adults’ and youths’ physical health [17, 24–26]. In addition, some studies highlight that owning a refrigerator reduces anxiety related to access to healthy food and stress related to the time required to procure food more frequently [27], as well as depression, embarrassment or discomfort [28]. However, the relevance of having a refrigerator for mental health is still often understudied.

### Air conditioning and mental health

Another health determinant recently proposed as essential is the presence of air conditioning in the home. As global temperatures continue to rise due to climate change, the use of air conditioning is becoming increasingly widespread [29]. However, huge differences related to air conditioning access characterize several world regions, and a growing divide between those who can afford this resource and those who cannot has been pointed out as the new indicator of inequality [30]. Access to air conditioning in the home has been shown to reduce heat-related mortality [31], increase productivity [32], and mitigate the adverse effects of high temperature on mental health, such as poorer concentration, elevated fatigue, aggressive behavior, stress, and anxiety [33, 34]. These results are promising and consistent with a recent review investigating the association between air conditioning and heat-related health outcomes [35]. However, only a small percentage of available studies focused on mental health, and, when they do, they often address adults’ mental health [36], with very few exploring the role of air conditioning on the mental health of children and adolescents. To the best of our knowledge, only one recent study has highlighted the mitigating role of air conditioning in reducing the adverse effects of heat on adolescents’ mental health [37], underscoring the need for further research on this topic.

### Domestic help and mental health

When exploring household living conditions, previous research has focused more on their physical aspects, such as access to water and owning appliances, while paying less attention to domestic help that represents another key dimension that might influence both physical and mental health [38]. The detrimental effects of housework tasks such as childcare, cleaning, cooking, and home maintenance on mental health have been pointed out for both women and youth, especially in low-resource settings [39, 40]. When household members perform these tasks, it is usually unpaid labor that requires significant energy and decreases time available for other activities such as school, paid work, and leisure (including social activities). This burden is especially pronounced in lower resource settings, where households are increasingly smaller and nuclear (therefore providing fewer helping hands) and institutional support is limited, therefore making domestic tasks highly time-consuming and especially stressful [41]. In these contexts, any instrumental support through paid help could be particularly beneficial and has been widely explored as a protective factor against mental health problems [42, 43]. However, despite the increasing acknowledgment of the burden of domestic work for both adults and youth, research on this topic remains limited in combination with other household living conditions [44, 45], making it especially important to study domestic help in conjunction with access to material resources.

### Why explore these specific household living conditions?

The public health relevance of examining household living conditions, such as access to a refrigerator, water, air conditioning, and domestic help, in relation to mental health lies in their similarities and shared role in supporting human development. These conditions ensure that all individuals live, develop, and thrive in a safe, clean, and adequate home environment. An environment with these characteristics aligns with the standard of living defined in Article 25 of the Universal Declaration of Human Rights, promotes individuals’ psychological well-being, and enables them to reach their full potential, in line with the Sustainable Development Goals. Indeed, the household living conditions examined in the current study constitute important everyday resources that influence individuals’ lived experiences and exposure to stress, as they directly affect daily routines, physical comfort, and the ability to meet basic needs. Living in a household with water access, a refrigerator, air conditioning and domestic help can buffer environmental and social stressors. Conversely, the absence of these conditions might act as chronic stressors. For instance, living without a refrigerator requires household members to get food every day, and significantly increases improper food storage, microbial growth, and bacterial contamination, leading to illness and malnutrition. This example shows how the lack of these conditions results in greater time demands in everyday household tasks and constant effort from household members to meet basic daily needs. As a direct consequence, youth in the household might find more difficulties in attending school, have less time to play and spend time with peers, and prematurely assume adult-like roles. All these aspects (i.e., lower school attendance, less playing and relational activities, and adultification) have detrimental effects on youths’ cognitive, emotional, and psychological development and interfere with children’s and adolescents’ right to thrive [46, 47]. Similarly, adults exposed to inadequate living conditions might face heightened concerns about physical safety and health, reduce the time available for working (therefore increasing financial instability), and have less capacity to provide consistent care for children or other social activities. All these aspects increase adults’ overall anxiety and depression and reduce opportunities for personal and social development [48].

### Household living conditions and mental health: the context of India and urban and rural differences

Although these aspects of household living conditions play a key role in advancing progress toward the Sustainable Development Goals, considerable regional differences and disparities both in the quality of living conditions and in mental health outcomes persist in several countries [49]. Among them, India represents a particularly relevant context. As one of the largest democracies in the world, India is undergoing consistent economic, scientific, and technological growth, but still exhibits high levels of poverty, food insecurity, malnutrition, and inadequate living conditions, alongside marked differences in living conditions between urban and rural households. In light of India’s demographic growth, the country is at a critical moment in restructuring its urban and rural systems, requiring local governments to promote sustainable and inclusive urbanization. However, few studies have been conducted in India, thereby requiring a deeper understanding of how household living conditions are associated with mental health across the life span in this context, with the ultimate goal to promote policies and reduce inequalities.

In the Indian socioeconomic context, household living conditions vary across regions. According to the most recent statistics [50], in India, nearly 95% of urban households have access to basic drinking water, compared to 91% of rural households. The gap becomes more pronounced when considering ownership of household appliances such as refrigerators and air conditioning. While 68% of urban households own a refrigerator, this is the case for only 33% of rural households. Similarly, air conditioning is available in 42% of urban households but in only 24% of rural ones. These numbers become particularly significant, given that recent studies have shown a substantial increase in temperatures and heat waves in India [51, 52]. Regarding domestic work and help, data on differences in access to domestic help between urban and rural households in India are limited. However, the characteristics of these areas are deeply connected to the current structure of domestic help in India. Specifically, the demand for paid domestic work has increased significantly among urban households over the last few decades, thereby prompting people to migrate from rural to urban areas to work as domestic workers [53]. This phenomenon explains why two-thirds of domestic workers reside in urban areas and why most of the domestic duties are performed in urban households by women, often migrating from marginalized communities and working in informal and underregulated environments, with low pay, poor job security, and no laws to protect their rights and health [41, 54]. Despite these well-documented structural vulnerabilities that clearly call for policy and labor protections, the presence of domestic help may be a way to reduce household stressors for family members. When domestic work is adequately regulated and carried out under fair and humane working conditions, it may contribute to alleviating caregiving and domestic burdens within households, thereby reducing mental health difficulties of household members.

Differences between urban and rural regions in India in the assessment and treatment of mental health have also been widely documented. Several studies underscored a higher prevalence of mental health issues in rural regions compared to urban ones, along with a shortage of mental health services and a stronger social stigma surrounding mental health problems [55, 56]. Beyond these regional differences, India is among the world regions where significant challenges persist in the detection and treatment of mental health problems, given that only a small percentage of individuals seek support for mental health problems [57, 58].

Taken together, all these aspects emphasize the need for further research aiming at informing policymakers on the needs of youth and adults living in urban and rural areas, identifying structural inequalities that affect access to basic health services, reducing possible disparities, and providing adequate services to specific stressors that youth and adults experience. This is particularly needed considering that, as far as we know, no studies have examined the connection between household living conditions and the mental health of youth and adults while also testing for differences between urban and rural regions.

### The current study

The current study builds on prior literature underscoring the role of adequate household living conditions (i.e., water access, refrigerator ownership, air conditioning, and domestic help) in supporting individuals’ health and aims to expand current knowledge on how such conditions influence the mental health (i.e., depression and anxiety) of youth and adults. At the same time, it examines whether there are differences between urban and rural regions in how household living conditions are related to mental health. These aims are directly related to SDG 3 (Good Health and Well-being), as they address mental health among youth and adults, as well as SDG 10 (Reduced Inequalities), and SDG 11 (Sustainable Cities and Communities), as they explore differences between urban and rural areas.

As for the association between household living conditions and mental health, we hypothesized that youth and adults living in a home with unlimited water access, a refrigerator, air conditioning, and domestic help would report better mental health (i.e., lower depression and anxiety scores). As for differences between urban and rural regions in the associations between household living conditions and mental health, we did not formulate specific hypotheses but instead adopted an exploratory approach, given the lack of prior research on whether these conditions are associated with mental health differently across urban and rural regions.

## Methods

### Study design

Participants were recruited in collaboration with the Centre for Monitoring Indian Economy (CMIE). Since 2014, CMIE has been conducting a longitudinal survey called the Indian Consumer Pyramids Household Survey (CPHS) [59]. Data are collected three times a year across 175,000 households and their 675,000 household members, covering a broad spectrum of economic and social indicators and employing geographic sampling to ensure the representativeness of 98.5% of India’s population. From February 2022 to June 2023, a subset of CPHS households was also asked to complete the Survey of Health Trends (SEHAT) module, with the overall goal to study trajectories of mental health in India, including children, adolescents, and adults of all age groups. This module was administered to households interviewed in the second month of each CPHS wave (February, June, and October), collecting data from individuals who were present at home at the time of the interview. The current analysis is based on mental health data collected in June 2023, which was selected as the most distant wave from the COVID-19 pandemic to minimize potential confounding effects of COVID-19 on mental health. Therefore, for this study, we used both secondary data on household living conditions and primary data on youths’ and adults’ mental health. Our sample comprises 8,205 households in rural regions and 19,979 households in urban regions. These households were composed of 29,188 members in rural regions and 67,351 members in urban regions. Among these household members, 37,453 adults provided information on their own depression and anxiety. In addition, 20,397 adults reported on the depression, and 19,363 on the anxiety, of youth living in the household. The research protocol was approved by [BLIND VERSION] (HMSC 2024–17967), [BLIND VERSION] IRB Protocol 2022 − 0113, [BLIND VERSION] (IMB-IRB2021#37), as well as [BLIND VERSION] (Project ID: 1827/009).

### Measures

*Youth mental health* was assessed using two parent-reported instruments. Depression symptoms were measured using the 5 items of the internalizing subscale from the Pediatric Symptoms Checklist (PSC) [60], asking adults to report how often youth in the household experienced depressive symptoms (e.g., *“Feels hopeless”*) on a 4-point scale from “never” to “always.” Anxiety symptoms were measured using the generalized anxiety subscale of the Spence Children’s Anxiety Scale (SCAS) [61], asking adults to report how often youth in the household experienced anxious symptoms (e.g., *“My child worries that something bad will happen to him/her”*) on a 4-point scale from “never” to “always.” For both children’s and adolescents’ depression and anxiety, a composite sum of all items was created (α = 0.71 for depression, α = 0.77 for anxiety). Higher scores indicated more youth depression and anxiety (range values for depression = 0–13; range values for anxiety = 0–16).

*Adults’ depression and anxiety* were assessed using two self-report questionnaires. Depression symptoms were measured using Patient Health Questionnaire-9 (PHQ-9) [62], asking adults to report how often in the past two weeks they experienced depressive symptoms (e.g., “*Little interest or pleasure in doing things*”) on a 4-point scale from “not at all” to “nearly every day.” Anxiety symptoms were measured using the Generalized Anxiety Disorder-7 (GAD-7) [63], asking adults to report how often in the past two weeks they experienced anxiety symptoms (e.g., “*Feeling nervous*,* anxious or on edge*”) on a 4-point scale from “not at all” to “nearly every day.” For both depression and anxiety, a composite sum of all items was created (α = 0.82 for depression, α = 0.81 for anxiety). Higher scores indicated more adult depression and anxiety (range values for depression = 0–27; range values for anxiety = 0–21).

*Household living conditions* include water access, refrigerator ownership, air conditioning, and domestic help. Data about refrigerator ownership, water access, and air conditioning were collected between January and April 2022; data about domestic help were collected in April 2023. For *water access*, respondents indicated whether the household had access to water within the household premises (via either a municipal water tap or a privately owned well), and how many hours per day water was accessible. We created a binary variable with 0 = limited access (i.e., no access to water within premises, or access not available every day of the week, or for fewer than 24 h per day) and 1 = unlimited access (i.e., access within the premises, available every day of the week and for 24 h every day). For *refrigerator ownership*, household members reported whether they owned a refrigerator and, if so, how many refrigerators were in the house. As fewer than 1% of participants reported owning more than one refrigerator, we created a binary variable (0 = no refrigerator in the household, 1 = refrigerator present in the household). For *air conditioning*, household members reported the number of air conditioners (i.e., air conditioners of various kinds, such as window ACs and split ACs) in the home. As fewer than 1% owned more than one air conditioner, we created a binary variable (0 = no air conditioning, 1 = air conditioning present). Finally, for *domestic help*, respondents reported monthly expenses related to laundry services and hired help (i.e., maid servants, cooks, drivers, guards, gardeners, babysitters, and other household staff). We created a binary variable with 0 = no domestic help, and 1 = yes domestic help present.

#### Other covariates

In each model, we also included the following control variables: adult respondent’s age and gender, caste, household education, number of people in the household (i.e., household size), household average income (in rupees), and the interaction between household average income and region type. Specifically, we categorized castes into scheduled caste (SC) and scheduled tribes (ST), other backward classes (OBC), intermediate caste, and upper caste (UC). Following CMIE recommendations, we created the household education measure by unifying literacy and education for all adults, categorizing it into low (no literate or educated adults), medium (some literacy and education), and high household education (including college graduates). Finally, we created the household average income by taking the mean of household incomes for the three months before data collection. In the models focused on child and adolescent depression and anxiety, we also included the young person’s age and gender as additional control variables.

### Data analyses

We estimated a series of linear mixed-effects models to examine the associations between household living conditions and the mental health of youth and adults in urban and rural regions. Specifically, we ran four separate models (i.e., two for the youths’ anxiety and depression, and two for the adults’ anxiety and depression). Each model included a random intercept to account for the nesting of participants within households, the fixed effects of living conditions (i.e., refrigerator ownership, water access, air conditioning, and domestic help), and the covariates (i.e., young person’s age and gender, adult respondent’s age and gender, caste, household education, number of people in the household, household average income, and the interaction between income and region type). To test for differences between urban and rural regions, we added interactions between the region types and all the household living conditions explored. All analyses incorporated survey weights at both the individual and household levels. We also ran four unadjusted linear mixed-effects models, omitting the covariates (Supplemental Tables 1 and 2 report the results), and obtained results that were consistent with those from the adjusted models. Moreover, as a sensitivity analysis, we tested for a dose–response relationship between living conditions and mental health outcomes. Specifically, to test whether youths’ and adults’ mental health outcomes improve as the number of favorable living condition indicators increases, we created a dosage variable by summing all household living conditions (0 = no living conditions present, 4 = all living conditions) and tested dosage associations with youths’ and adults’ mental health. We used R software version 4.2.2 [64] and the packages lme4 [65], lmerTest [66], and WeMix [67] to perform all statistical analyses. Missing data were handled using complete-case analysis (i.e., listwise deletion under default package options).

## Results

### Household living conditions, mental health, and regional differences: descriptive analyses

Table 1 reports the descriptive analyses. In both urban and rural regions, youth were mainly males (57%) between the ages of 5 and 19 years old (mean age = 13.90 in urban regions and 13.12 in rural regions), adult reporters were mainly females (83% and 79% in urban and rural regions) with a mean age of 46 years, and households were composed of an average of 4 members. In urban regions, most households (43.2%) were highly educated (i.e., including college graduates), whereas in rural regions, most households (43.6%) were low in education (i.e., no literate or educated adults). As for mental health, youths’ depression and anxiety were higher in rural regions compared to urban regions. Interestingly, adults’ depression and anxiety followed the opposite trend and were higher in urban regions compared to rural ones. To further explore these comparisons, we performed a series of one-way ANOVAs, which showed a statistically significant difference in youths’ depression between urban and rural regions (F (1, 84) = 12.69, *p* < .001), but no difference in youths’ anxiety scores (F (1, 15) = 1.55, *p* = .22). Conversely, both adults’ depression and anxiety were significantly different in urban and rural regions (F (1, 628) = 24.3, *p* < .001; F (1,223) = 11.78, *p* < .001, respectively). While these results underscore urban and rural differences, it is important to note that overall levels of depression and anxiety in our sample were low. Supplemental Table 3 reports detailed descriptive statistics about youths’ and adults’ mental health. Looking at the distribution of scores and considering the established cutoffs to indicate potential anxiety and depression disorders reported in the literature, our participants fell below these cutoffs.

Regarding household living conditions, the most pronounced difference between urban and rural regions was related to refrigerator ownership, with 74.5% of households in urban regions owning a refrigerator, compared to only 54.5% of households in rural regions. Similarly, air conditioning and domestic help were more commonly reported in urban regions, whereas water access showed minimal difference between regions, with approximately 75% of households in both rural and urban areas reporting unlimited water access.

**Table 1:** Descriptive analyses divided by urban and rural regions

	Urban regions (*N* = 67,351)	Rural regions (*N* = 29,188)
	M (SD) or *N* (%)	M (SD) or *N* (%)
Mental Health		
Youths’ Depression	2.86 (2.52)	2.99 (2.69)
Youths’ Anxiety	3.36 (2.98)	3.41 (3.23)
Adults’ Depression	5.88 (5.07)	5.59 (5.12)
Adults’ Anxiety	4.82 (4.35)	4.64 (4.36)
Household living conditions		
Water access		
Limited	10,356 (25%)	4,304 (24.17%)
Unlimited	31,198 (75%)	13,506 (75.83%)
Refrigerator ownership		
No	14,264 (25.2%)	9,911 (45.5%)
Yes	42,352 (74.8%)	11,855 (54.5%)
Air conditioning		
No	50,048 (88.4%)	21,436 (98%)
Yes	6,568 (11.6%)	330 (2%)
Domestic help		
No	56,448 (83.8%)	27,518 (94.3%)
Yes	10,903 (16.2%)	1,670 (5.7%)
Control variables		
Young person age (5–19)	13.90 (4.37)	13.12 (4.61)
Young person females	6,075 (43%)	3,045 (43%)
Adult respondent age	46.53 (11.36)	45.74 (11.49)
Adult respondent females	56,132 (83%)	23,149 (79%)
Not stated caste	861 (1.5%)	206 (1%)
Scheduled caste/scheduled tribes	12,896 (21.7%)	7,933 (33.1%)
Other backward classes	23,092 (38.8%)	10,001 (41.8%)
Intermediate caste	5,246 (8.8%)	2,784 (11.6%)
Upper caste	17,365 (29.2%)	2,995 (12.5%)
Low household education	16,451 (27.7%)	10,420 (43.6%)
Medium household education	17,290 (29.1%)	8,035 (33.6%)
High household education	25,708 (43.2%)	5,456 (22.8%)
Household size (1–15)	3.97 (1.42)	4.22 (1.56)
Household average income	27,947 (20,572)	22,941 (18,239)

* N reports household members

### Household living conditions, mental health, and regional differences: linear mixed-effects models

Tables 2 and 3 show the results of the four linear mixed-effects models with random intercepts for household effects. Considering the multilevel nature of our data, we examined the within- and between-household variance and relations between our predictors and outcomes. In every model, most variance was between households and not within households. The unconditional intraclass correlation coefficients (ICCs), the proportion of variance that is due to between-household differences before adding predictors, were 0.87 for youths’ depression, 0.89 for youths’ anxiety, 0.81 for adults’ depression, and 0.82 for adults’ anxiety. After accounting for covariates, the adjusted ICCs were 0.85 for youths’ depression, 0.88 for youths’ anxiety, 0.84 for adults’ depression, and 0.84 for adults’ anxiety, suggesting a strong clustering effect in every model.

**Table 2:** Linear mixed-effects models with random intercepts for household effects: youths’ mental health

	Youths’ Depression		Youths’ Anxiety	
	B (SE)	*p*	B (SE)	*p*
Water access	-1.34 (0.14)	**< 0.001**	-1.24 (0.18)	**< 0.001**
Refrigerator ownership	− 0.43 (0.12)	**< 0.001**	− 0.51 (0.14)	**< 0.001**
Air conditioning	0.54 (0.47)	0.25	1.02 (0.62)	0.10
Domestic help	-1.09 (0.19)	**< 0.001**	− 0.99 (0.22)	**< 0.001**
Urban region	0.40 (0.18)	**0.03**	0.64 (0.24)	**0.006**
Water access x Urban region	− 0.06 (0.17)	0.71	− 0.28 (0.21)	0.17
Refrigerator ownership x Urban region	0.08 (0.14)	0.58	0.21 (0.17)	0.23
Air conditioning x Urban region	-1.21 (0.49)	**0.013**	-2.00 (0.65)	**0.002**
Domestic help x Urban region	1.05 (0.23)	**< 0.001**	1.00 (0.27)	**0.003**

Unstandardized (B) estimates, standard errors, and *p*-values are reported in the table. Unstandardized estimates are reported due to the binary nature of the data. Water access, refrigerator, and air conditioning ownership, and domestic help were coded as 0 = no, 1 = yes. Region types were coded as 0 = rural, 1 = urban. The model controlled for young people’s age and gender, adult respondents’ age and gender, castes, household education, household size, average income, and interaction between average income and region type

Bolded *p*-values are *p*<.05

**Table 3:** Linear mixed-effects models with random intercepts for household effects: adults’ mental health

	Adults’ Depression		Adults’ Anxiety	
	B (SE)	*p*	B (SE)	*p*
Water access	-1.55 (0.17)	**< 0.001**	-1.23 (0.15)	**< 0.001**
Refrigerator ownership	-1.05 (0.16)	**< 0.001**	− 0.79 (0.14)	**< 0.001**
Air conditioning	0.48 (0.78)	0.54	0.67 (0.59)	0.25
Domestic help	-1.13 (0.27)	**< 0.001**	− 0.77 (0.30)	**< 0.001**
Urban region	0.94 (0.24)	**< 0.001**	1.07 (0.20)	**< 0.001**
Water access x Urban region	− 0.77 (0.21)	**< 0.001**	− 0.93 (0.18)	**< 0.001**
Refrigerator ownership x Urban region	0.59 (0.19)	**0.002**	0.49 (0.17)	**0.003**
Air conditioning x Urban region	-1.64 (0.81)	**0.041**	-1.62 (0.61)	.**008**
Domestic help x Urban region	1.42 (0.32)	**< 0.001**	1.15 (0.26)	**< 0.001**

Unstandardized (B) estimates, standard errors, and *p*-values are reported in the table. Unstandardized estimates are reported due to the binary nature of the data. Water access, refrigerator, and air conditioning ownership, and domestic help were coded as 0 = no, 1 = yes. Region types were coded as 0 = rural, 1 = urban. The model controlled for adult respondents’ age and gender, castes, household education, household size, and average income, and interaction between average income and region type

Bolded *p*-values are *p*<.05

### Children’s and adolescents’ mental health

Several significant moderation effects by region type emerged in relation to youths’ mental health. Regarding water access and refrigerator ownership, the association between these factors and youth mental health was observed in both urban and rural regions. Specifically, water access and refrigerator ownership were associated with lower mental health symptom scores in both rural and urban regions (Table 2). However, the association was stronger in urban regions (depression: B = -1.40, *p*<.001; anxiety: B = -1.52, *p*<.001) compared to rural regions (depression: B = -1.34, *p*<.001; anxiety: B = -1.24, *p*<.001) for water access. This pattern was further supported by joint tests of the main and interaction terms, which indicated that the association between water access and mental health was significantly different from zero in urban regions (depression: Wald χ²(1) = 253.4, *p* < .001; anxiety: Wald χ²(1) = 180.65, *p* < .001). Conversely, the association between refrigerator ownership and mental health was weaker in urban regions (depression: B = − 0.35, *p* < .001; anxiety: B = − 0.30, *p*<.001) compared to rural regions (depression: B = − 0.43, *p* < .001; anxiety: B = − 0.51, *p* = .05). This pattern was further supported by joint tests of the main and interaction terms, which indicated that the association between refrigerator ownership and mental health was significantly different from zero in urban regions (depression: Wald χ²(1) = 16.242, *p* < .001; anxiety: Wald χ²(1) = 7.8044, *p* = .005).

Regarding air conditioning, no associations with mental health (depression: B = 0.54, *p*=.025; anxiety: B = 1.02, *p* = .10) were observed in rural regions, whereas air conditioning was linked to lower depression (B = − 0.67, *p*<.001) and anxiety (B = − 0.97, *p* < .001) in urban regions. Joint tests of the main and interaction terms indicated that the association between refrigerator ownership and mental health was significantly different from zero in urban regions (depression: Wald χ²(1) = 19.378, *p* < .001; anxiety: Wald χ²(1) = 24.324, *p* < .001). Finally, the availability of domestic help was associated with lower depression among youth living in rural regions (B = -1.09, *p*<.001) and not in urban ones (B = − 0.03, *p*=.81), as well as lower anxiety only for youth living in rural regions (B = − 0.99, *p*<.001), with no evidence of an association in urban regions (B = 0.01, *p* = .95). This pattern was further supported by joint tests of the main and interaction terms, which indicated that the association between domestic help and both depression and anxiety was not significantly different from zero in urban regions (depression: Wald χ²(1) = 0.0604, *p* = .805; anxiety: Wald χ²(1) = 0.0041, *p* = .949).

### Adults’ mental health

Regarding adults’ mental health, water access was associated with lower depression and anxiety scores in both rural and urban regions (Table 3). However, the association was stronger in urban regions (depression: B = -2.32, *p*<.001; anxiety: B = -2.16, *p*<.001) than in rural regions (depression: B = -1.55, *p*<.001; anxiety: B = -1.23, *p*<.001). A joint test of the main and interaction terms indicated that the association between water access and mental health was significantly different from zero in urban regions (depression: Wald χ²(1) = 382.76, *p* < .001; anxiety: Wald χ²(1) = 491.17, *p* < .001).

Regarding refrigerator ownership, owning a refrigerator was associated with lower depression and anxiety scores in both rural and urban regions. However, the association was stronger in rural regions (depression: B = -1.05, *p*<.001; anxiety: B = − 0.79, *p*<.001) than in urban regions (depression: B = − 0.45, *p*<.001; anxiety: B = − 0.29, *p* = .003). These patterns were further supported by a joint test of the main and interaction terms, which indicated that the association between refrigerator ownership and adults’ mental health was significantly different from zero in urban regions (depression: Wald χ²(1) = 14.482, *p* < .001; anxiety: Wald χ²(1) = 8.7461, *p* = .003).

We discovered a significant difference between urban and rural regions in the association between air conditioning and depression and anxiety. Specifically, air conditioning was significantly associated with lower depression and anxiety scores only in urban regions (depression: B = -1.16, *p* < .001; anxiety: B = − 0.95, *p*<.001) and not in rural ones (depression: B = 0.48, *p* = .54; anxiety: B = 0.67, *p* = .25). These patterns were further supported by a joint test of the main and interaction terms, which indicated that the association between refrigerator ownership and adults’ mental health was significantly different from zero in urban regions (depression: Wald χ²(1) = 32.899, *p* < .001; anxiety: Wald χ²(1) = 30.717, *p* < .001).

Finally, domestic help was not associated with adults’ depression in urban regions (B = 0.29, *p* = .07), whereas it was related to lower adults’ depression in rural regions (B = − 0.13, *p*<.001). It was also associated with lower adult anxiety scores in rural regions (B = − 0.77, *p*<.001) compared to urban ones (B = 0.37, *p* = .006). These patterns were further supported by a joint test of the main and interaction terms, which indicated that the association between domestic help and adults’ mental health was significantly different from zero in urban regions only for anxiety (Wald χ²(1) = 7.7122, *p* = .006) and not for depression (Wald χ²(1) = 3.3492, *p* = .07).

### Sensitivity analyses

Supplemental Tables 4 and 5 report the results of the dose-response analyses. For both youths’ and adults’ mental health, our findings showed a dose–response relationship between household living conditions and mental health outcomes. As for youth, each additional living condition that was present in the household was associated with a decrease in depression and anxiety scores (depression: B = − 0.61, *p*<.001; anxiety: B = − 0.64, *p*<.001). These associations were significantly attenuated in urban regions (depression: B = 0.26, *p* < .001; anxiety: B = 0.28, *p*<.001). As for adults, each additional living condition that was present in the household was associated with a decrease in depression and anxiety scores (depression: B = -1.11, *P*<.001; anxiety: B = − 0.93, *p*<.001). This association was significantly attenuated in urban regions (depression: B = 0.36, *p*<.001; anxiety: B = 0.32, *p*<.001).

## Discussion

Although extensive evidence indicates the association between adequate household living conditions and individuals’ physical health worldwide [8, 9], relatively few studies have been conducted on how specific household living conditions, such as water access, refrigerator ownership, air conditioning, and domestic help are associated with youths’ and adults’ depression and anxiety, especially looking at urban and rural differences and at Indian context. The current study draws on a large and nationally representative sample of both urban and rural regions in India to expand the current knowledge on this topic.

### Youths’ and adults’ mental health: urban and rural differences

Looking at the descriptive analyses of overall urban and rural differences in mental health (without considering the effects of household living conditions), our findings indicate that youth living in rural regions reported higher scores on depression compared to those living in urban regions. This significant difference may be attributed to the specific challenges that young individuals face in rural areas, including geographical isolation, longer travel distances to access services and facilities, inadequate mental health services, as well as fewer educational, cultural, and leisure opportunities [68]. Interestingly, the opposite trend emerged among adults, with those living in urban regions reporting more depression and anxiety than their rural counterparts. This apparently contradictory result may reflect psychosocial stressors associated with living in urban environments, such as traffic congestion, air and noise pollution, population density and crowding, housing problems, and socioeconomic inequality —factors that are linked to poor mental health outcomes [69]. Taken together, our findings suggest that environmental challenges associated with urban and rural settings may have age-specific effects on mental health, rather than being inherently better or worse for individuals, with distinct influences depending on individuals’ developmental tasks and life stages.

### Youths’ and adults’ mental health and household living conditions

Exploring the effects of household living conditions on youths’ and adults’ mental health, we discovered that, in both urban and rural regions, unlimited water access, as well as ownership of a refrigerator, were associated with lower depression and anxiety in youth and adults. The positive effects of these living conditions can be attributed to their numerous benefits for everyday life, as widely highlighted in the literature [23, 25]. Overall, living in a house where water access is guaranteed and prevents diseases, and a refrigerator ensures safe food storage and impedes food contamination, provides individuals with more hygienic living conditions and a greater sense of stability and security. Consequently, these living conditions reduce physical and mental burdens and stressors in everyday life for household members, thereby allowing individuals to thrive, spending more time and energy attending school or work, or developing their own potential, ultimately leading to lower anxiety and depression.

Regarding urban and rural differences in the associations between household living conditions and mental health, the first regional difference concerned the association between water access and mental health. Specifically, water access appeared to be more strongly associated with lower depression and anxiety among youth and adults living in urban areas than for their rural counterparts. This pattern may reflect different overall access to water in urban and rural settings, with water availability being more uncertain or costly in cities than in rural regions, mostly due to infrastructure limitations, services interruptions, or heat-related problems. These aspects may hinder consistent and secure access to water, thereby negatively impacting mental health [23]. Conversely, for youth and adults residing in rural regions, refrigerator ownership appeared to be more strongly associated with lower depression and anxiety scores, whereas individuals living in urban regions seem to benefit less from refrigerator ownership. A possible explanation for this pattern may be that urban settings offer greater and more diverse access to food options, thereby reducing individuals’ reliance on a refrigerator. Thanks to the widespread availability of restaurants, grocery stores, and take-out services, urban residents are more likely to have continuous access to a wide variety of food, which is often processed, less perishable, and readily available. These conditions may foster a greater sense of food security and, consequently, reduce anxiety scores compared to individuals living in contexts where food availability is more challenging. Conversely, individuals living in rural regions may encounter more difficulties in accessing food options, such as having to travel long distances to purchase food or having fewer restaurants in their area [70, 71].

Additionally, for youth and adults living in urban regions, air conditioning appeared to be linked with lower depression and anxiety scores compared to their counterparts in rural regions. These patterns suggest that air conditioning may offer protective effects against heat-related stress and adverse mental health outcomes primarily in urban contexts. Compared to rural settings, urban areas are characterized by high levels of pollution, extensive paved surfaces, limited green spaces, housing structures retaining more heat, and population density [72, 73], characteristics that, combined, contribute to the ‘urban heat island’ (UHI) phenomenon, a situation when temperatures in urban areas are substantially higher than in rural regions that are located nearby [74].

Finally, for youth and adults living in rural regions, the availability of domestic help was more protective against mental health problems compared to individuals living in urban regions. Possible explanations for this difference may be that individuals living in rural regions face more burdensome, hazardous, and physically demanding housework activities compared to those in urban settings. Furthermore, in rural areas, individuals may have access to fewer services that facilitate household tasks compared to urban settings, and a traditional division of labor where women and youth are primarily responsible for domestic work may persist [75].

### Strengths, limitations, and implications of the current study

This study has several strengths. First, it draws on a large, frequently assessed, and nationally representative sample of households from all states in India, encompassing a broad spectrum of economic and social indicators and specific associations between the selected living conditions and mental health. Although the effect sizes were small – a common risk when working with large datasets – our findings provided valuable insights and revealed interesting associations between household living conditions and mental health among youth and adults, as well as urban-rural differences in these associations. These findings provide a valuable contribution to a topic and context that remain underrepresented in research. Second, we controlled for factors such as caste, income, and household education to increase the rigor of the analyses.

Although these strengths are highly valuable, the current study comes with several limitations. First, even though we used data collected at different time points, the data are correlational and do not enable causal inferences. Future research would benefit from collecting longitudinal data to track changes in household living conditions and mental health associations over time and across different developmental periods. Longitudinal data would also be needed to explore potential mechanisms underlying the observed associations, using mediation analyses. For instance, although we included household education and income as covariates, future studies might explore the role of a range of factors that might mediate the associations between living conditions and the mental health of youth and adults. Second, we created binary variables to explore household living conditions, which limits our ability to examine nuanced aspects related to these conditions that may be linked to the mental health of youth and adults. Third, although participants were asked about appliance ownership, we did not collect data about the actual use, purpose, source of securing and functioning of those appliances. Future studies might explore whether and how these aspects moderate the associations between household living conditions and mental health. Fourth, in the current study, we did not explore other variables that might be important for assessing the association between household living conditions and mental health, such as access to toilets or characteristics of walls and roofs. Future directions would benefit from integrating all these living conditions to create a more comprehensive composite, allowing for a full investigation of living conditions across urban and rural regions, as well as their implication for the mental health of children, adolescents, and adults. Fifth, although we controlled for gender and socioeconomic factors, our primary focus was on exploring differences in the associations between household living conditions and mental health across urban and rural regions. Future studies might build on this by investigating whether these associations vary by gender and socioeconomic status. Finally, given that refrigerator ownership, air conditioning and domestic help are highly correlated with household income, these variables might be considered, at least in part, as proxies for household socioeconomic status. While our models controlled for household average income, level of education, and the interaction between average income and region type, it is possible that this control may not fully account for all dimensions of socioeconomic status. Therefore, our findings should be interpreted with caution, as they may reflect not only isolated effects of living conditions but also broader economic resources and household socioeconomic differences. Future studies might benefit from exploring this aspect more by testing combinations of these living conditions with additional forms of economic resources.

Despite all these limitations, the current findings have several practical implications. First, as household living conditions (i.e., water access, refrigerator ownership, air conditioning, and availability of domestic help) were associated with the mental health of individuals across age groups, it is recommended to incorporate household-level environmental assessments into mental health screening, particularly in low- and middle-income settings. This would allow clinicians and community health workers to increase their information to better contextualize mental health symptoms and guide more tailored interventions. Second, the findings support a preventive, public health-oriented approach to mental health, suggesting that interventions aimed at improving household living conditions might benefit individuals’ mental health, too. Third, the differences between urban and rural regions suggest that interventions should be context-sensitive, responding to specific needs in each context, both in terms of living conditions and mental health. Finally, India has often been underrepresented in research, despite being characterized by structural inequalities, big differences between urban and rural regions, and significant challenges in detecting mental health problems. Therefore, the current findings represent an important opportunity to expand knowledge of the Indian context, with significant implications for developing more integrated care models for both youth and adults.

## Conclusions

Although living in an adequate home environment is essential to promote the psychological health of children, adolescents, and adults, several studies have examined the adverse effects of inadequate household living conditions on physical health, devoting less attention to mental health. The current study advances understanding of the associations between specific living conditions of the household (i.e., water access, refrigerator ownership, air conditioning, domestic help) and youths’ and adults’ mental health, by using a large and nationally representative sample of both urban and rural regions in India. The current study yields several key conclusions. First, across different age groups, accessing water, having a refrigerator, air conditioning and domestic help were linked with lower depression and anxiety scores. However, significant differences emerged between urban and rural regions. Indeed, having a refrigerator and domestic help were related to lower mental health problems especially when individuals live in rural settings compared to urban ones. Conversely, access to water and air conditioning was connected to lower depression and anxiety especially in urban settings. These findings underscore the connection between these living conditions and mental well-being, suggesting that living conditions may represent a cost-effective, yet often overlooked avenue that should be taken into account to design mental health interventions.

## Supplementary Information

Supplementary Material 1.

## Data Availability

The SEHAT module of the dataset used and/or analyzed during the current study is available from the corresponding author upon reasonable request. The CPHS dataset is available with a subscription from the Centre for Monitoring Indian Economy Pvt.Ltd (http://www.cmie.com).
