# Supplementary material for: Household living conditions and mental health of youth and adults: differences between urban and rural regions
Source: BMC Psychol. 2026 May 30;14:1124. doi: 10.1186/s40359-026-04906-7 (PMC13430773; doi:10.1186/s40359-026-04906-7)
Supplement: Supplementary file 1 — Supplementary Material 1. [file 40359_2026_4906_MOESM1_ESM.docx]

**Supplemental Table 1**

Unadjusted linear mixed-effects models with random intercepts for household effects and no control variables included: Youths’ depression and anxiety.

|  | Youths’ Depression | | Youths’ Anxiety | |
| --- | --- | --- | --- | --- |
|  | B (SE) | *p* | B (SE) | *p* |
| Water access | -1.30 (.13) | **< .001** | -1.13 (.17) | **< .001** |
| Refrigerator ownership | -.39 (.10) | **< .001** | -.48 (.13) | **< .001** |
| Air conditioning | .69 (.47) | .14 | 1.10 (.60) | .06 |
| Domestic help | -.93 (.17) | **< .001** | -1.08 (.19) | **< .001** |
| Urban region | .18 (.15) | .24 | .33 (.19) | .09 |
| Water access x Urban region | -.03 (.16) | .84 | -.27 (.20) | .16 |
| Refrigerator ownership x Urban region | .12 (.13) | .37 | .24 (.16) | **.13** |
| Air conditioning x Urban region | -1.13 (.50) | **.024** | -1.70 (.63) | **.007** |
| Domestic help x Urban region | 1.01 (.21) | **< .001** | 1.25 (.25) | **<.001** |

**Supplemental Table 2**

Unadjusted linear mixed-effects models with random intercepts for household effects and no control variables included: Adults’ depression and anxiety.

|  | Adults’ Depression | | Adults’ Anxiety | |
| --- | --- | --- | --- | --- |
|  | B (*SE*) | *p* | B (*SE*) | *p* |
| Water access | -1.46 (.17) | **< .001** | -1.12 (.14) | **< .001** |
| Refrigerator ownership | -.82 (.14) | **< .001** | -.55 (.12) | **< .001** |
| Air conditioning | 1.09 (.79) | .17 | 1.19 (.63) | .07 |
| Domestic help | -1.22 (.24) | **< .001** | -.74 (.21) | **<.001** |
| Urban region | .76 (.19) | **<.001** | .80 (.17) | **< .001** |
| Water access x Urban region | -.77 (.20) | **< .001** | -.95 (.17) | **< .001** |
| Refrigerator ownership x Urban region | .40 (.18) | **.025** | .31 (.15) | **.039** |
| Air conditioning x Urban region | -2.02 (.81) | **.013** | -1.87 (.65) | **.004** |
| Domestic help x Urban region | 1.55 (.28) | **<.001** | 1.18 (.24) | **<.001** |

**Supplemental Table 3**

Detailed descriptive statistics for outcome variables

|  |  | **Urban regions**  (N = 67,351) | | | **Rural regions**  (N = 29,188) | | |
| --- | --- | --- | --- | --- | --- | --- | --- |
|  | Cutoff for potential disorder | M (SD) | Median | Min-Max | M (SD) | Median | Min-Max |
| Youths’ Depression – 5 items from PSC | ≥ 5 | 2.86 (2.52) | 2.00 | 0-13 | 2.99 (2.69) | 3.00 | 0-13 |
| Youths’ Anxiety – Generalized Anxiety Scale of SCAS | ≥ 7 | 3.36 (2.98) | 3.00 | 0-16 | 3.41 (3.23) | 3.00 | 0-15 |
| Adults’ Depression – PHQ-9 | ≥5 mild  ≥10 moderate | 5.88 (5.07) | 4.00 | 0-27 | 5.59 (5.12) | 4.00 | 0-24 |
| Adults’ Anxiety – GAD-7 | ≥5 mild  ≥10 moderate | 4.82 (4.35) | 4.00 | 1-21 | 4.64 (4.36) | 4.00 | 0-20 |

*Note.* For youths’ mental health, cutoffs are reported for the subscales of the Internalizing scale of the Pediatric Symptoms Checklist, for the Generalized Anxiety Scale of the Spense Children’s Anxiety Scale (SCAS)

**Supplemental Table 4**

Sensitivity analyses: Dose-response analyses with youths’ depression and anxiety

|  | Youths’ Depression | | Youths’ Anxiety | |
| --- | --- | --- | --- | --- |
|  | B (SE) | *p* | B (SE) | *p* |
| Dosage household living conditions | -.61 (.06) | **< .001** | -.64 (.07) | **< .001** |
| Urban region | -.33 (.16) | **.038** | -.33 (.20) | .11 |
| Dosage household living conditions x Urban region | .26 (.07) | **<.001** | .28 (.08) | <.001 |

*Note.* Region types were coded as 0 = rural, 1 = urban. The model controlled for young people’s gender and age, adult respondents’ age and gender, castes, household education, household size, and average income, and interaction between average income and region type.

**Supplemental Table 5**

Sensitivity analyses: Dose-response analyses with adults’ depression and anxiety

|  | Adults’ Depression | | Adults’ Anxiety | |
| --- | --- | --- | --- | --- |
|  | B (SE) | *p* | B (SE) | *p* |
| Dosage household living conditions | -1.11 (.08) | **< .001** | -.93 (.07) | **< .001** |
| Urban region | .13 (.21) | .54 | -.08 (.18) | .54 |
| Dosage household living conditions x Urban region | .36 (.09) | <.001 | .32 (.08) | <.001 |

*Note.* Region types were coded as 0 = rural, 1 = urban. The model controlled for adult respondents’ age and gender, castes, household education, household size, and average income, and interaction between average income and region type.
